# Acetamide Derivatives with Antioxidant Activity and Potential Anti-Inflammatory Activity

**DOI:** 10.3390/molecules15032028

**Published:** 2010-03-23

**Authors:** Giuseppina Autore, Anna Caruso, Stefania Marzocco, Barbara Nicolaus, Chiara Palladino, Aldo Pinto, Ada Popolo, Maria S. Sinicropi, Giuseppina Tommonaro, Carmela Saturnino

**Affiliations:** 1 Department of Pharmaceutical Sciences, University of Salerno, Fisciano (SA), Italy; E-Mails: autore@unisa.it (G.A.); caruso.anna@inwind.it (A.C.); smarzocco@unisa.it (S.M.); chiara24@hotmail.it (C.P.); pintoal@unisa.it (A.P.); apopolo@unisa.it (A.P.); 2 Institute of Biomolecular Chemistry, CNR, Pozzuoli (NA), Italy; E-Mails: gtommonaro@icmib.na.cnr.it (G.T.); bnicolaus@icmib.na.cnr.it (B.N.); 3 Department of Pharmaceutical Sciences, University of Cosenza, Arcavacate di Rende (CS), Italy; E-Mail: s.sinicropi@unical.it (M-S.S.)

**Keywords:** amides, antioxidant, antiproliferative activity, brine shrimp test

## Abstract

This study reports the synthesis and antioxidant activity of some new acetamide derivatives. The compounds’ structures were elucidated by NMR analysis and their melting points were measured. The *in vitro* antioxidant activity of these compounds was tested by evaluating the amount of scavenged ABTS radical and estimating ROS and NO production in tBOH- or LPS-stimulated J774.A1 macrophages. All compounds were tested for their effect on cell viability by an MTT assay and by a Brine Shrimp Test.

## Introduction

**Scheme 1:**
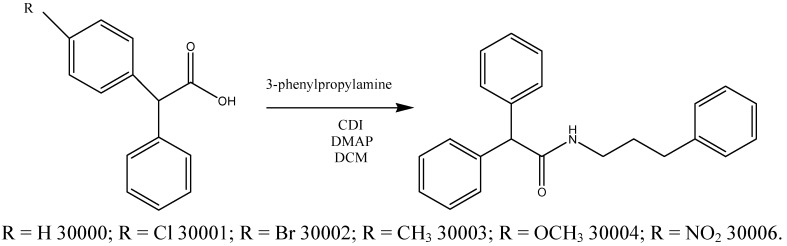
Synthesis of compounds **30000-4** and **6**.

The antioxidant activity is related with compounds capable of protecting a biological system against the potential harmful effects of oxidative processes. In the last years antioxidant compounds have received increased attention from nutritionists and medical researchers for their potential activities in preventing cancer, cardiovascular disorders, as well as aging [1]. Several papers have been published on structure-activities analysis of new antioxidant compounds. In this study we report for the first time the synthesis and the *in vitro* antinflammatory and antioxidant activities of some new acetamide derivatives. The syntheses of the compounds **30000-4** and **6** (as reported in Scheme 1) were accomplished using 2-phenylacetic acid derivatives (1 eq) dissolved in dry dichloromethane (DCM, 10 mL). To this solution were added *N,N^1^*-carbonyldiimidazole (CDI, 2 eq) and 4-dimethylaminopyridine (DMAP, 2 eq) and the mixture was stirred for 20 minutes at rt. After an appropriate 3-phenyl-propylamine (2 eq) was added and the mixture further stirred for 5 h at rt.

**Scheme 2:**
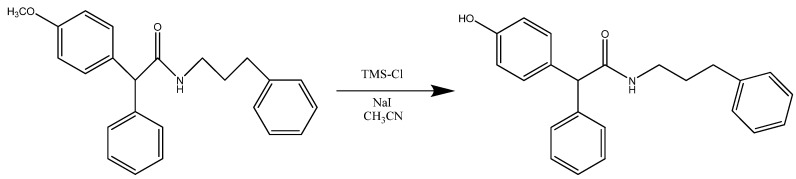
Synthesis of 2-(4-hydroxyphenyl)-2-phenyl-*N*-(3-phenylpropyl)acetamide (**30005**).

The synthesis of compound **30005** (Scheme 2) was achieved using 2-(4-methoxyphenyl)-2-phenyl-*N*-(3-phenylpropyl)acetamide (1 eq) and sodium iodide (2.2 eq) dissolved in acetonitrile (15 mL). Chlorotrimethylsilane (2.2 eq) was added to the solution which was stirred for 16 h at reflux. 

**Scheme 3:**
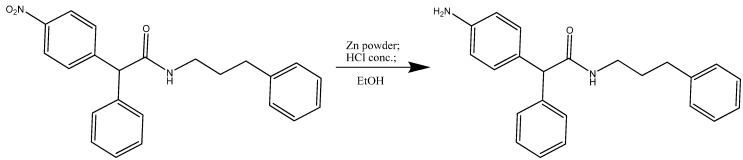
Synthesis of 2-(aminophenyl)-2-phenyl-*N*-(3-phenylpropyl)acetamide (**30007**).

The synthesis of compound **30007** (Scheme 3) was accomplished using 2-(4-nitrophenyl)-2-phenyl-*N*-(3-phenylpropyl) acetamide (1 eq) and zinc powder (10 eq) dissolved in EtOH (10 mL). Conc. HCl (1 eq) was added to the solution and cooled to -10 °C. 

**Scheme 4:**
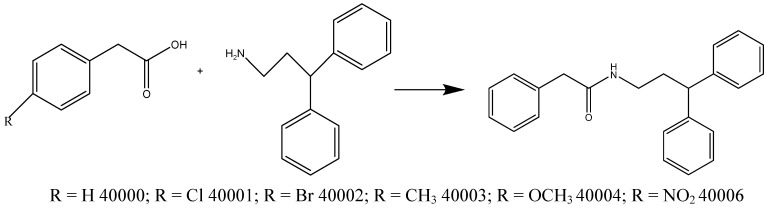
Synthesis of compounds **40000-4** and **6**.

The synthesis of the compounds **40000-4** and **6** (Scheme 4) were done using 2-phenylacetic acid derivatives (1 eq) dissolved in dry DCM (10 mL); afterwards CDI (2 eq) and DMAP (0.5 eq) were added to the solution that was stirred for 20 minutes at rt. Thereafter was added 3,3-diphenylpropan-1-amine (1 eq) and the mixture stirred for 24 h at rt. 

**Scheme 5:**
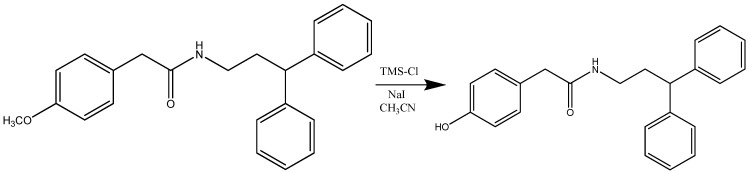
Synthesis of *N*-(3,3-diphenylpropyl)-2-(4-hydroxyphenyl)acetamide (**40005**).

The synthesis of compound **40005** (Scheme 5) was achieved using *N*-(3,3-diphenylpropyl)-2-(4-methoxyphenyl)acetamide (1 eq) and sodium iodide (3 eq) dissolved in acetonitrile (35 mL). To the solution heated to 23 °C was added chlorotrimethylsilane (2.2 eq) and the mixture was stirred for 16 h at reflux. 

**Scheme 6:**
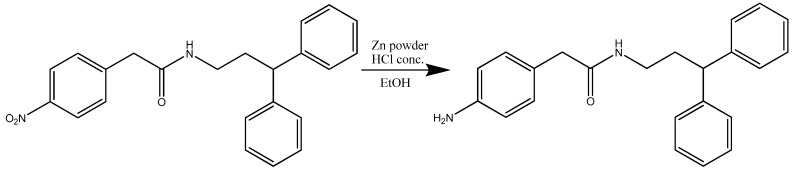
Synthesis of 2-(4-aminophenyl)-*N*-(3,3-diphenylpropyl) acetamide (**40007**).

The synthesis of compound **40007** (Scheme 6) was accomplished using *N*-(3,3-diphenylpropyl)-2-(4-nitrophenyl) acetamide (1 eq) and zinc powder (10 eq) dissolved in EtOH (40 mL). Conc. HCl (1 eq) was added to the solution and it was cooled to -10 °C. 

## Results and Discussion

The brine shrimp lethality bioassay is an efficient, rapid and inexpensive test developed as a prescreen to evaluate biological activities of natural or synthetic compounds. In Table 1 we report the biological activity of the samples in the brine shrimp assay, expressed as LD_50_ (in ppm). All compounds showed good activity in this test, in particular compounds **40006** and **40007**, which showed interesting LD_50 _values of 3.0443 and 10.6444 ppm, respectively. None of the newly synthesized compounds were cytotoxic on macrophage cell line J774.A1, as revealed by a MTT test (Table 2). Among the tested compounds we choose to further investigate the pharmacological activity of **40006** and **40007** because of their good results in the ABTS and brine shrimp test. LPS increased NO production in the medium of J774.A1 macrophages, addition to the culture medium of graded concentration of 40006 and 40007 (0.1–10 µM) 1 h before and simultaneously to LPS significantly (*P* < 0.001) reduced NO production (Table 3).

**Table 1:** Biological activity of compounds **30003-30007** and **40003-40007**. The experiments were performed in triplicate.

SAMPLES	% INHIBITION ABTS^.+^	T.E.A.C. (μM)	LD_50_ (ppm)
			[95% confidence intervals]
**30003**	1%	0.26	n.a.
**30004**	2%	0.52	n.a.
**30005**	0.8%	0.20	n.a.
**30006**	1%	0.26	41.4591
			[84.3018/23.5460]
**30007**	10.6%	2.74	29.8785
			[45.6981/19.8115]
**40003**	n.a.	/	58.3200
			[132.3034/32.7936]
**40004**	2%	0.52	17.8661
			[28.9967/11.1125]
**40005**	5.1%	1.32	17.0035
			[28.5947/10.2798]
**40006**	4%	1.03	3.0443
			[4.7830/1.9283]
**40007**	26.2%	6.77	10.6444
			[16.8533/6.6340]

**Table 2:** Shows the results, expressed as IC50 (M) values, indicating the concentration of each compound that affords cell growth by 50% as compared to control cells. n.d. = not detectable.

	Cell lines		
Compounds	HEK-293	WHEI-164	J774.A1
**30003**	n.d.	n.d.	n.d.
**30004**	1.067 × 10^-4^	1.790 × 10^-6^	n.d.
**30005**	4.170 × 10^-5^	2.235 ×x 10^-6^	n.d..
**30006**	2.439 × 10^-6^	1.913 × 10^-4^	n.d..
**30007**	n.d.	1.513 × 10^-4^	n.d.
**40003**	2.5 × 10^-4^	2.441 × 10^-6^	> 10^-4^
**40004**	n.d.	n.d.	2.324 × 10^-4^
**40005**	1.665 × 10^-4^	1.038 × 10^-6^	> 10^-4^
**40006**	> 10^-4^	2.05 × 10^-5^	2.324 × 10^-4^
**40007**	2.069 × 10^-5^	4.124 × 10^-5^	> 10^-4^

**Table 3:** Effect of compounds **40006** and **40007** on nitrite production by LPS- induced J774.A1 macrophages. Results are expressed as mean ± S.E.M. % inhibition calculated versus nitrite produced by J774.A1 treated with LPS alone of at least 3 independents experiments with three replicates each. Comparison was performed using one way analysis of variance. ** P < 0.01 *vs.* LPS.

	% inhibition nitrite production *vs* LPS		
Compounds	10 µM	1 μM	0.1 μM
**40006**	79.66 ± 1.33**	86.75 ± 0.65**	91.73 ± 1.59 **
**40007**	85.00 ± 3.51**	89.6 ± 1.46**	81.5 ± 12.67 **

**Figure 1:**
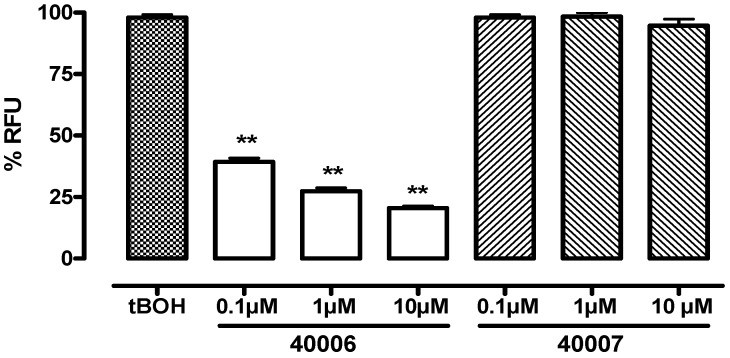
Effect of compounds **40006** and **40007** on ROS production by tBOH-induced J774.A1 macrophages. ROS reaction was determined by measuring the converting reaction of DCFH_2 _to DCF. ROS production were expressed as mean ± S.E.M. of relative fluorescent units (RFU) of at least three independent experiments. ** P < 0.01 *vs.* tBOH.

As reported in Figure 1, compound **40006** also significantly (P < 0.01) reduced ROS production in J774.A1 macrophages stimulated with *terz-*butyl hyproperoxide (tBOH), while compound **40007** have no effect on ROS inhibition.

## Experimental

### General

All reagents were purchased from Sigma-Aldrich s.r.l. (Milan, Italy). The reactions were monitored by thin-layer chromatography (TLC), using Whatman K6F silica gel on aluminum and alumina (Merck) plates with fluorescence indicators and appropriate solvents. A >95% purity could be inferred from the ^1^H-NMR spectra. Melting points were taken on a Gallenkamp melting point apparatus and are uncorrected. The organic extracts were dried over sodium sulphate dry (Merck). ^1^H-NMR spectra were recorded with a Bruker Advance 300 MHz spectrometer, using CDCl_3_ as solvent. Mass spectrometry analysis ESI-MS was cwrried out on a Finningan LCQ Deca ion trap instrument.

### Synthesis of compounds ***30000-4*** and ***6***

An appropriate acid was dissolved in dry DCM (10 mL). To this solution were added *N,N^1^*-carbonyldiimidazole (CDI, 0.612 g; 3.78 mmol; FW = 162.15; 2 eq) and 4-dimethylaminopyridine (DMAP, 0.115 g; 0.94 mmol; FW = 122.17; 2 eq) and the resulting mixture was stirred for 20 minutes at rt. After 3-phenylpropylamine (0.511 g; 3.78 mmol; FW = 135.21; 2 eq) was added and the mixture stirred for 5 h at rt [2]. To the mixture was added H_2_SO_4 _(2N) and it was extracted with ethyl acetate. The organic layer was dried with anhydrous sodium sulphate, filtered and the solvent removed by evaporation. The residue was purified by column chromatography on silica (Pe/EtOAc 6:4;Pe/EtOAc 4:6). After recrystallization from DCM/Pe, the products were obtained as white crystals 

*Compound* **30000**: Yield: 0.390 g (64%); Melting point: 135 °C; MS: m/z 331 (M^+ ^+ 2); 329 (M^+^); ^1^H-NMR δ 8.0 (s, 1H), 7.39-6.94 (m, 15H), 5.58-5.43 (s, 1H), 3.53-3.30 (t, 2H), 2.68-2.56 (t, 2H), 1.89-1.77 (q, 2H). 

*Compound* **30001**: Yield: 0.650 g (70%); Melting point: 125 °C; MS: [M] = 363.9; [M +H^+^] = 364.9; ^1^H-NMR δ 7.39-7.04 (m, 14 H), 5.60-5.53 (s, 1H), 4.88 (s, 1H), 3.53-3.37 (q, 2H), 2.68-2.60 (t, 2H), 1.89-1.77 (q, 2H

*Compound* **30002**: Yield: 0.210 g (81%); Melting point: 135 °C; MS: [M – H^+^] = 407.1; [M + H^+^] = 409.1; ^1^H-NMR δ 7.50-7.46 (d, 2H), 7.37-7.10 (m, 12 H), 5.57 (s, 1 H), 4.48 (s, 1 H), 3.40-3.30 (q, 2H), 2.64-2.59 (t, 2H), 1.96-1.82 (p, 2H). 

*Compound* **30003**: Yield: 0.640 g (71%); Melting point: 110 °C; MS: (M) 342.2, (m + H^+^) 345.2; ^1^H-NMR δ 7.62-7.21 (m, 12 H), 7.18-7.10 (d, 2 H), 5.57 (s, 1 H), 4.90 (s, 1H), 3.38-3.30 (q, 2 H), 2.63-2.57 (t, 2 H), 1.88-1.77 (p, 2 H). 

*Compound* **30004**: Yield: 0.640 g (78%); Melting point: 110 °C; MS: [M] = 359.5; [M+H^+^] = 360.5; ^1^H-NMR δ 7.64-7.20 (m, 12H), 6.92 (d, 2H), 5.58 (s, 1H), 4.88 (s, 1 H), 3.87 (s, 3 H), 3.40-3.30 (q, 2 H), 2.64-3.57 (t, 2 H), 1.89-1.76 (p, 2 H). 

*Compound* **30006**: Yield: 0.600 g (78%); Melting point: 117 °C; MS: [M] = 374.4; [M +H^+^] = 375.4; ^1^H-NMR δ 8.40 (d, 2H); 7.70 (d, 2H); 7.45-7.10 (m, 10H); 5.5 (s, 1H); 4.87 (s, 1H); 3.92-3.85 (t, 2H); 3.35-3.28 (q, 2H); 2.34-2.22 (q, 2H).

### Synthesis of compounds ***30005*** and ***30007***

2-(4-Methoxyphenyl)-2-phenyl-N-(3-phenylpropyl)acetamide (0.480 g; 1.34 mmol; FW = 359.5; 1 eq) and sodium iodide (0.440 g; 2.95 mmol; FW = 149.89; 2.2 eq) were dissolved in acetonitrile (15 mL). Chlorotrimethylsilane (377 μL; 2.95 mmol; FW = 108.64; d = 0.850; 2.2 eq) was added to the solution and stirred for 16 h at reflux. The mixture was poured into water and extracted with ethyl acetate, the organic layer was washed with Na_2_S_3_O_3_ and Brine, dried with anhydrous sodium sulphate, filtered and evaporated. The residue products were purified by column chromatography on silica (Pe/EtOAc 8:2; Pe/EtOAc 7:3). After recrystallization with DCM/Pe , white crystals were obtained (0.310 g; 66%). Melting point: 85 °C. m/z 347.2 (M^+^+ 2); 345.2 (M^+^). ^1^H NMR δ 8.80-7.18 (m, 10H); 7.10-7.00 (d, 2H); 6.90-6.77 (d, 2H); 5.67 (s, 1H); 4.82 (s, 1H); 3.89-3.80 (t, 1H); 3.33-3.24 (q, 2H); 2.30-2.20 (q, 2H) (**30005**) .

2-(4-nitrophenyl)-2-phenyl-N-(3-phenylpropyl) acetamide (0.200 g; 0.53 mmol; FW = 450.5; 1 eq) and zinc powder (0.346 g; 5.3 mmol; FW = 63.93; 10 eq) were dissolved in EtOH (10 mL). HCl conc. (3 mL; 0.53 mmol; FW = 36.46; 1 eq) was added to the solution and cooled to -10 °C. The mixture was stirred and heated at 80 °C for 3h. After was poured into water and NaOH to neutralize the excess of acid, the organic layer was extracted with Et_2_O, dried with anhydrous sodium sulphate, filtered and removed by evaporation. The residue products were purified by column chromatography on silica (Pe/EtOAc 1:1). A yellow-orange oil was obtained (0.160 g; 88%). m/z 346.4 (M^+^+ 2); 344.4 (M^+^). ^1^H NMR (CDCl_3_, 300 Mhz) δ 7.37-7.19 (m, 8H); 7.15 (d, 2H); 7.05 (d, 2H), 6.68 (d, 2H); 5.59 (s, 1H); 4.48 (s, 1H); 3.68 (s, 2H); 3.36 (q, 2H); 2.63 (t, 2H); 2.10 (p, 2H) (**30007**).

### Synthesis of compounds ***40000-4*** and ***40006***

An appropriate acid was dissolved in DCM dry (10 mL) and N^1^-carbonyldiimidazole CDI (0.541 g; 4.43 mmol; FW = 162.15; 2 eq) and 4-dimethylaminopyridin DMAP (0.134; 1.10 mmol; FW = 122.17; 0.5 eq) were added to the solution and stirred for 20 minutes at rt. Thereafter was added 3,3-diphenylpropan-1-amine (0.936 g; 4.43 mmol; FW = 211.3; 1 eq) and stirred for 24h at rt. To the mixture was added H_2_SO_4 _(2N) and extracted with ethyl acetate. The organic layer was dried with anhydrous sodium sulphate, filtered and removed by evaporation. The residue products were purified by column chromatography on silica (Pe/EtOAc 9:1; Pe/EtOAc 8:2; Pe/EtOAc 7:3; Pe/EtOAc 6:4). After recrystallization with DCM/Pe, white crystals were obtained (0.620 g; 85%). [M] = 329; [M+H^+^] = 330. ^1^H NMR (CDCl3, 300 Mhz) δ 8.0 (s, 1H), 7.41-7.06 (m, 15H), 4.10-4.05 (t, 1H), 3.44 (s, 2H), 3.33-3.20 (t, 2H), 2.19-2.11 (q, 2H) (**40000**).

After recrystallization with DCM/Pe, white crystals were obtained (0.750 g; 71%). [M] = 363; [M+H^+^] = 364. ^1^H NMR ( CDCl_3_, 300 Mhz) δ 7.64-6.94 (m, 14H), 5.27 (s, 1H), 3.91 (t, 1H), 3.47 (s, 2H), 3.27-3.16 (t, 2H), 2.29-2.18 (q, 2H) (**40001**).

After recrystallization with DCM/Pe, white crystals were obtained (0.700 g; 75%). [M] = 407; [M + H^+^] = 408. ^1^H NMR (CDCl_3_, 300 Mhz) β 7.80 (d, 2H), 7.32-7.12 (m, 10H), 6.96 (d, 2H), 5.33 (s, 1H), 3.90 (t, 1H), 3.46 (s, 2H), 3.27-3.16 (t, 2H), 2.29-2.19 (q, 2H) (**40002**).

After recrystallization with DCM/Pe, white crystals were obtained (0.930 g; 82%). Melting point: 110 °C. [M] = 343.5; [M+H^+^] = 344.5. ^1^H NMR (CDCl_3_, 300 Mhz) δ 7.30-7.16 (m, 14H), 5.30 (s, 1H), 3.87 (t, 1H), 3.50 (s, 2H), 3.21 (q, 2H), 2.25 (q, 2H) (**40003**).

After recrystallization with DCM/Pe, white crystals were obtained (80%). [M] = 343.5; [M+H^+^] = 344.5. ^1^H NMR (CDCl_3_, 300 Mhz) δ 7.30- 7.16 (m, 14H), 5.30 (s, 1H), 3.87 (t, 1H), 3.50 (s, 2H), 3.21 (q, 2H), 2.25 (q, 2H) (**40004**).

After recrystallization with DCM/Pe, white crystals were obtained (80%). [M] = 343.5; [M + H^+^] = 344.5. ^1^H NMR (CDCl_3_, 300 Mhz) δ 7.30-7.16 (m, 14H), 5.30 (s, 1H), 3.87 (t, 1H), 3.50 (s, 2H), 3.21 (q, 2H), 2.25 (q, 2H) (**40006**).

### Synthesis of compounds ***40005*** and ***40007***

N-(3,3-diphenylpropyl)-2-(4-methoxyphenyl)acetamide (1.2 g; 3.4 mmol; FW = 359.5; 1 eq) and sodium iodide (1.5 g; 10.2 mmol; FW = 149.89; 3 eq) were dissolved in acetonitrile (35 mL). To the solution heated to 23 °C was added chlortrimethylsilane (1.1 mL; 10.2 mmol; FW = 108.64; d = 0.850; 2.2 eq) and stirred for 16h at reflux. The mixture was poured into water and extracted with ethoxyethane, the organic layer was washed with Na_2_S_3_O_3_ and Brine, dried with anhydrous sodium sulphate, filtered and evaporated. The residue products were purified by column chromatography on silica (Pe/EtOAc 8:2; Pe/EtOAc 7:3; Pe/EtOAc 6:4). After recrystallization with DCM/Pe, white crystals were obtained (0.510 g; 45%). Melting point: 85 °C. m/z 347.2 (M^+^+ 2); 345.2 (M^+^). ^1^H NMR (CDCl_3_, 300 Mhz) δ 8.80-7.18 (m, 10H), 7.10-7.00 (d, 2H), 6.90-6.77 (d, 2H), 5.67 (s, 1H), 4.82 (a, 1H), 3.89-3.80 (t, 1H), 3.33-3.24 (q, 2H), 2.30-2.20 (q, 2H) (**40005**).

N-(3,3-diphenylpropyl)-2-(4-nitrophenyl) acetamide (1.00 g; 2.67 mmol; FW = 374.4; 1 eq) and zinc powder (1.75 g; 26.7 mmol; FW = 65.41; 10 eq) were dissolved in EtOH (40 mL). HCl conc. (5 mL; 0.53 mmol; FW = 36.46; 1 eq) was added to the solution and cooled to -10 °C. The mixture was stirred and heated at 80 °C for 3h. Thereafter was poured into water and NaOH to neutralize the excess of acid, the organic layer was extracted with Et_2_O, dried with anhydrous sodium sulphate, filtered and removed by evaporation. The residue products were purified by column chromatography on silica (Pe/EtOAc 1:1). Was obtained a yellow-orange oil (0.900 g; 97%). m/z 346.4 (M^+^ + 2); 344.4 (M^+^). ^1^H NMR (CDCl_3_, 300 Mhz), δ 7.37-7.19 (d, 2H), 7.15 (d, 2H), 7.05 (m, 8H), 6.68 (d, 2H), 5.59 (s, 1H), 4.84 (s, 1H), 3.68 (s, 2H), 3.36 (q, 2H), 2.63 (t, 2H), 2.10 (q, 2H) (**40007**).

### Antioxidant activity assay by ABTS method

The antioxidant activity of compounds was determined by ABTS method, as previously described [3,4]. This method is based on the capacity of different components to scavenge the ABTS radical cation (ABTS^•^+) compared to standard antioxidant (Trolox) in dose response curves. Briefly ABTS^•^+ radical cation is obtained by reacting ABTS (7 mM) with potassium persulphate; this mixture was stored in the dark for 12-16 h before use. Before the assay, the mixture was diluted in ethanol at a ratio 1:100 to give an absorbance at λ = 734 nm of 0.70 + 0.02. TROLOX was used as standard at a concentration of 1 mg/mL and aliquots of Trolox (0.5 μL, 1 μL, 2 μL, 3 μL, 5 μL and 10 μL ) were added to 1 mL of ethanolic ABTS^•^+ to have a standard curve to which all data are referred. All compounds were dissolved in dichloromethane at a concentration of 20 mg/mL and 5 μL were added to ethanolic ABTS^•^+ to measure absorbance after 1 min. 

Antioxidant activity was carried out in triplicate and expressed as percentage of the absorbance of the uninhibited radical solution according to the equation:

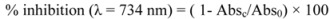

where Abs_0_ was the absorbance of uninhibited radical solution and Abs_c_ is the absorbance measured 1 min after addition of compound to assay. The antioxidant activity of samples was expressed also as T.E.A.C. (Trolox Equivalent Antioxidant Capacity - μM) [5].

### Brine Shrimps Test

The brine shrimps (*Artemia salina*) assay was performed in triplicate with appropriate amounts of samples dissolved in DMSO to reach final concentrations of 1, 10 and 100 ppm, using 10 freshly hatched larvae suspended in 5 mL of artificial sea water [6]. Briefly, for each dose tested, surviving shrimps were counted after 24 h, and the data analyzed by the Finney program [7], which affords LD_50_ values with 95% confidence intervals.

### Analysis of NO_2-_ Production

The murine macrophage cell line, J774.A1, was obtained from American Tissue Culture Collection (ATCC). J774.A1 cells were maintained in DMEM supplemented with NaHCO_3_ (42 mM), hepes (25 mM), penicillin (100 units/mL), streptomycin (100 units/mL), glutamine (2 mM) and foetal calf serum (FCS, 10%) at 37 °C in a 95% air and 5% CO_2_ atmosphere.

J774.A1 (3.5 × 10^4^ cells/well) were plated on 96-well microtiter plates and allowed to adhere at 37 °C in a 5% CO_2_ atmosphere for 2 h. To stimulate the expression of the inducible form of nitric oxide synthase (iNOS), *E. coli* lipopolysaccharide (LPS, 6 × 10^3^ u/mL) was added to fresh culture medium of J774.A1 with graded concentrations of tested compounds (1–100 μg/mL) added 1h before and simultaneously with LPS challenge. Nitric oxide release, evaluated as nitrite (NO_2_^-^) accumulation in cell culture medium, was evaluated 24 h after LPS stimulation by Griess reagent [8].

### MTT Assay for Antiproliferative Activity

Murine macrophage cells (J774.A1) were maintained as previously indicated. Murine fibrosarcoma cells (WEHI-164) were maintained in adhesion on Petri dishes with DMEM supplemented with 10% heat-inactivated FCS, hepes (25 mM), penicillin (100 u/mL) and streptomycin (100 μg/mL). Human embryonic kidney cells (HEK-293) were maintained and grown in adhesion on Petri dishes with DMEM supplemented with FCS (10 %), hepes (25 mM), penicillin (100 u/mL) and streptomycin (100 units/mL).

J774.A1, WEHI-164 and HEK-293 (3.5 × 10^4^ cells/well) were plated on 96-well microtiter plates and allowed to adhere at 37 °C in a 5 % CO_2_ atmosphere for 2 h. Thereafter, the medium was replaced with fresh one (50 μL) and a 75 μL aliquot of each tested compound was added and then the cells incubated for further 72 h. 6-mercaptopurine was used as reference drug. Mitochondrial respiration, an indicator of cell viability, was assessed by the mitochondrial-dependent reduction of [3-(4,5-dimethylthiazol-2-yl)-2,5-phenyl-2H-tetrazolium bromide] (MTT) to formazan and cells viability was assessed accordingly to the method of Mosmann [9]. Briefly 5 μL of MTT (5 mg/mL) were added and the cells were incubated for an additional 3 h. Thereafter, cells were lysed and the dark blue crystals solubilised with 100 μL of a solution containing 50% (v:v) N, N-dimethylformamide, 20 % (w:v) SDS with an adjusted pH of 4.5 [10]. The optical density (OD) of each well was measured with a microplate spectrophotometer (Titertek Multiskan MCC/340) equipped with a 620 nm filter. The viability of each cell line in response to treatment with tested compounds and 6-mercaptopurine was calculated as: % dead cells = 100−(OD treated/OD control) × 100.

### Measurement of Reactive Oxygen Species (ROS)

The accumulation of ROS was evaluated by means of the probe 2',7'-dichlorofluorescein (DCF) according to the method described by Le Bel *et al*. [11]. Briefly, J774.A1 cells were seeded at a density of 5 × 10^3^ cells/well into 96-well plates and allowed to adhere for 2 h. After cell adhesion, examined compounds (1-100 μg/mL) were added to the culture medium 24 h before and always simultaneously to dichlorofluorescein-diacetate. 2',7'-Dichlorofluorescein-diacetate (H_2_DCF-DA, Sigma) was added directly to the growth medium at a final concentration of 5 µM and the cells incubated for 1 h at 37 °C. H_2_DCF-DA is a non-fluorescent permeant molecule which diffuses passively into cells; the acetates are then cleaved by intracellular esterases to form H_2_DCF which is thereby trapped within the cell. In the presence of intracellular ROS, H_2_DCF is rapidly oxidized to the highly fluorescent DCF. Therefore, cells were washed twice with phosphate-buffered saline (PBS), placed in fresh medium and treated with *terz-*butyl hyproperoxide (tBOH) 3 mM for 30 min and than were placed in a fluorescent microplate reader (LS 55 Luminescence Spectrometer; Perkin Elmer, Beaconsfield, Bucks, UK). Fluorescence was monitored using an excitation wavelength of 490 nm and an emission wavelength of 520 nm. Results were expressed as Relative Fluorescence Units (RFU).

## Conclusions

Results show that these acetamide derivatives, obtained by simple and rapid chemical synthesis, had interesting biological activities and that none of the compounds are toxic on J774.A1, the cell line chosen for the other tests. Among the tested compounds, **40006** showed the more interesting activity because of its inhibition of nitrite and ROS. Taken together, our data let us hypothesized that the inhibitory effect of compound **40006** on *t*-BuOH-induced NO formation in J774.A1 macrophages could be due to its free radical scavenger properties. Moreover, to have an antioxidant activity, is possible to modify the aromatic function binding to the amidic group, instead is necessary to keep unvaried the aromatic function binding to the alkylic chain. In fact the compound **40006** is more active than the compound **30006**. Further studies need to characterize its pharmacological activity.
